# Use of unstructured text in prognostic clinical prediction models: a systematic review

**DOI:** 10.1093/jamia/ocac058

**Published:** 2022-04-27

**Authors:** Tom M Seinen, Egill A Fridgeirsson, Solomon Ioannou, Daniel Jeannetot, Luis H John, Jan A Kors, Aniek F Markus, Victor Pera, Alexandros Rekkas, Ross D Williams, Cynthia Yang, Erik M van Mulligen, Peter R Rijnbeek

**Affiliations:** Department of Medical Informatics, Erasmus University Medical Center, Rotterdam, The Netherlands; Department of Medical Informatics, Erasmus University Medical Center, Rotterdam, The Netherlands; Department of Medical Informatics, Erasmus University Medical Center, Rotterdam, The Netherlands; Department of Medical Informatics, Erasmus University Medical Center, Rotterdam, The Netherlands; Department of Medical Informatics, Erasmus University Medical Center, Rotterdam, The Netherlands; Department of Medical Informatics, Erasmus University Medical Center, Rotterdam, The Netherlands; Department of Medical Informatics, Erasmus University Medical Center, Rotterdam, The Netherlands; Department of Medical Informatics, Erasmus University Medical Center, Rotterdam, The Netherlands; Department of Medical Informatics, Erasmus University Medical Center, Rotterdam, The Netherlands; Department of Medical Informatics, Erasmus University Medical Center, Rotterdam, The Netherlands; Department of Medical Informatics, Erasmus University Medical Center, Rotterdam, The Netherlands; Department of Medical Informatics, Erasmus University Medical Center, Rotterdam, The Netherlands; Department of Medical Informatics, Erasmus University Medical Center, Rotterdam, The Netherlands

**Keywords:** clinical prediction model, prognostic prediction, natural language processing, machine learning, electronic health records

## Abstract

**Objective:**

This systematic review aims to assess how information from unstructured text is used to develop and validate clinical prognostic prediction models. We summarize the prediction problems and methodological landscape and determine whether using text data in addition to more commonly used structured data improves the prediction performance.

**Materials and Methods:**

We searched Embase, MEDLINE, Web of Science, and Google Scholar to identify studies that developed prognostic prediction models using information extracted from unstructured text in a data-driven manner, published in the period from January 2005 to March 2021. Data items were extracted, analyzed, and a meta-analysis of the model performance was carried out to assess the added value of text to structured-data models.

**Results:**

We identified 126 studies that described 145 clinical prediction problems. Combining text and structured data improved model performance, compared with using only text or only structured data. In these studies, a wide variety of dense and sparse numeric text representations were combined with both deep learning and more traditional machine learning methods. External validation, public availability, and attention for the explainability of the developed models were limited.

**Conclusion:**

The use of unstructured text in the development of prognostic prediction models has been found beneficial in addition to structured data in most studies. The text data are source of valuable information for prediction model development and should not be neglected. We suggest a future focus on explainability and external validation of the developed models, promoting robust and trustworthy prediction models in clinical practice.

## INTRODUCTION

**Figure 1.**
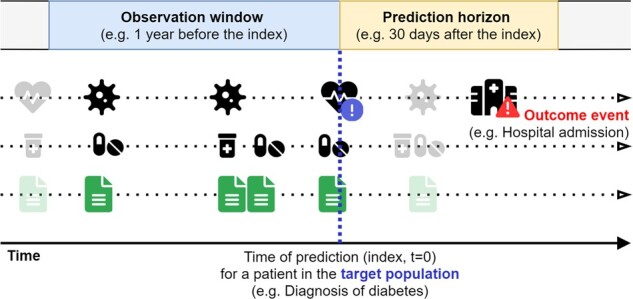
Visualization of the prognostic prediction problem. The objective is to predict which patients from a target population will experience an outcome event within a prediction horizon, using predictors only measured in an observation window before the time of prediction. Predictors can be extracted from both the structured data and text data.

Prognostic prediction models are increasingly common in clinical research and practice.^1^^,^^2^ Prognostic models predict which patients, among a target population of patients, will experience some clinical outcome during a window of time in the future, the prediction horizon. The predictors used by the model are measured during an observation window prior to the time of prediction (Figure 1). The growing availability of observational data in electronic health records (EHRs) forms a rich source to develop prediction models in a data-driven manner.^2^^,^^3^ Although most clinical risk prediction research is centered on the use of structured data, such as coded conditions, measurements, and drug prescriptions, the majority of information in EHRs is typically stored in vast quantities of unstructured text, for example, nursing notes, discharge letters, or radiology reports.^4^ When compared with structured data, unstructured text lacks an organized structure or terminology, is large in terms of file size, and contains patient-sensitive information, which complicates its use for the construction of prediction models. However, information captured in text can be more detailed and extensive than in structured data, as it is not limited to specific code systems or input fields. Therefore, the use of text data in a prediction model could potentially provide information to better predict the outcome, improving model performance.

The growing availability of unstructured text in EHR data, increased computational power, and progress in natural language processing (NLP) techniques are now enabling the use of text data for the development of prediction models. Several reviews have elucidated the use of text data in the clinical domain, focusing on the general task of extracting information from unstructured text^5–11^ or the diagnostic classification of patients, including case detection, patient identification, and phenotyping.^4^^,^^9^^,^^12^ However, the development of text-based prognostic prediction has not been extensively studied. Recently, Yang et al^13^ performed a large review of 579 prognostic prediction models, expanding on the review by Goldstein et al,^2^ but neither focused on the use of text data. Another review, by Yan et al^14^ studied the use of unstructured text in, specifically, early sepsis prediction. To our knowledge, no broad systematic review has been conducted on the development of text-based prognostic prediction models. As these models start to be developed, it becomes increasingly important to reflect on the work that has been done, summarize the methodological landscape, and discover whether text data have value supplementing structured data.

Consequently, the objective of this review is to assess how information extracted from unstructured text in EHR data is utilized to develop and validate prognostic prediction models. We evaluated the studies on the study settings and populations, text processing methods and representations, machine learning methods and feature set combinations, performance evaluation and external validation, attention to model explainability, and model availability. Furthermore, we determined the value of text in addition to structured data by comparing the performance between models using different feature sets within the studies.

## MATERIALS AND METHODS

### Review protocol

This study followed the *Preferred Reporting Items for Systematic Reviews and Meta-Analyses* (PRISMA).^15^ The review protocol was registered on June 17, 2021, and is publicly available at the Open Science Framework Registries (https://osf.io/gw628).

### Eligibility criteria

This review targeted studies from the last 15 years (January 2005 to March 2021) describing the development and evaluation of prognostic clinical prediction models that incorporate information extracted in a data-driven manner from unstructured text in EHR data. A range of 15 years was chosen to allow for a broad search, including early studies developing prediction models in a data-driven manner. The 3 inclusion criteria are defined as follows. (1) The study described the development and evaluation of a prognostic clinical prediction model. (2) The model predictors were based on information extracted from unstructured text in an EHR database. (3) Information was automatically extracted from the unstructured text in a data-driven manner. Data-driven implies that the extraction of information from the text was exploratory and not restricted to features that were expected to be important. This allowed us to evaluate prediction models developed on all the available text, comparable to the exploratory development of prediction models on all structured data, instead of a limited set of concepts. Detailed inclusion criteria are provided in Table 1.

**Table 1.** Inclusion criteria

Criterium		Description	Exclude examples
1		The study described the development and evaluation of a prognostic clinical prediction model	
	A	The model predicts a future clinical event or outcome for a patient	Exclude diagnostic, identification, phenotyping, or extraction models
	B	The subject must be a patient or a limited group of patients	Exclude if the subject is anything else, such as a drug, bed, or gene
	C	A parameterized prediction model must be developed and evaluated	Exclude if a study only reports the odd-ratios of covariates, only runs statistical tests, or does not evaluate the developed model
	D	Any clinical domain is relevant, such as intensive, radiology, general practitioner, or psychiatric care	
2		The model predictors were based on information extracted from unstructured text in an EHR database	
	A	The information is extracted from human-readable text data in an EHR database	Exclude if all text data comes from other sources, such as social media, literature, recordings, transcripts, or genetic data
	B	The extracted information is used as covariates in the model	Exclude when the study only uses the information to define the outcome or target patient cohorts
	C	The model must at least use information from unstructured text, but a combination with structured data is allowed	
3		Information was automatically extracted from the unstructured text in a data-driven manner	
	A	Data-driven means that the extraction is exploratory and it should not have been known beforehand what information from the text data was important for model development	Exclude if the extraction was driven by mere intuition, personal experience, or existing knowledge. For example, the extraction of a limited number or specific set of clinical concepts, such as the smoking status or a small set of vital signs
	B	The extraction was done automatically	Exclude if the information is manually extracted from the text data

### Literature search

Four databases were used for the literature search: Embase, MEDLINE, the Web of Science core collection, and Google Scholar. The database choice and the search strategy creation were aided by a medical librarian. The search strategy consisted of 4 clauses that incrementally limited the search results: (1) Prediction models; (2) The medical domain or EHRs; (3) A notion of text data, clinical notes, or NLP methods; (4) The period from January 2005 till March 2021, studies in the English language, and excluding conference abstracts and animal research. The full search strategy can be found in Supplementary Table S1.

### Screening

The found studies were first screened for fulfilling the eligibility criteria based on the title and abstract. Those that were found relevant underwent a second screening for inclusion based on the full text. In both screening phases, 1 reviewer (TS) screened all studies and 10 other reviewers (EF, SI, DJ, LJ, JK, AM, VP, AR, RW, and CY) independently screened 1/10th of the total number of studies. This resulted in each study being screened by 2 independent reviewers. Any discrepancies between them, in both screening phases, were resolved in a consensus meeting.

### Study quality assessment

To assess the quality of the studies 1 reviewer (TS) determined their adherence to the *transparent reporting of a multivariable prediction model for individual prognosis or diagnosis* (TRIPOD) statement^16^ using the TRIPOD adherence form (https://www.tripod-statement.org/adherence/).

### Data extraction and synthesis

Data for analysis were extracted from the included studies by 1 reviewer (TS) using a predefined set of data items, outlined in Table 2. Some items are based on clinical prediction item sets from the *critical appraisal and data extraction for systematic reviews of prediction modeling studies* (CHARMS) checklist^17^ and the TRIPOD statement.^16^ Ten data item topics were distinguished: (1) general publication information, (2) study setting, (3) study population, (4) unstructured text predictors, (5) structured data predictors, (6) machine learning methods and feature sets, (7) internal and (8) external validation, (9) model explainability, and (10) model availability.

**Table 2.** List of data items for data extraction, by topic

Item topic	Data item	Input type
1. General information	Publication year	Year
	Journal	Free text
2. Study setting	Dataset	Free text
	Country of data	Country
	Clinical setting	Free text
	Study dates	Range of years
3. Population	Type of study*	Cohort, case-control
	Target population^AB^	Free text
	Prediction outcome^A^	Free text
	Prediction horizon^A^	Hours, days, years, relative time (free text), and timepoint
	Prediction outcome type	Binary, multi-class, and continuous
4. Unstructured text predictors	Type of unstructured text	Free text
	Language of text	Language
	Observation window^B^	Hours, days, years, relative time (free text), and timepoint
	Preprocessing methods	Free text
	Text representation methods	*BoW*, TFIDF, *CE*, *WE*, *DE*, *TM*, and *SS* (multiple possible)
	Used ontologies/vocabularies	Free text
	Used software/program/package	Free text
	Number of predictors^A^	Number
5. Structured data predictors	Types of structured data	Free text
	Observation window^B^	Hours, days, years, relative time (free text), and timepoint
	Number of predictors^A^	Number
6. Model	Machine learning method^A^	*LogR*, *LinR*, *Cox*, *NB*, *RFTB*, *GB*, *SVM*, feedforward *NN*, *RNN*, *CNN*, transformers, *DNN*, ensembles, and other
	Feature set	Structured, text, and combined
7. Internal validation	Number of observations^A^	Number
	Number of observations with the outcome (outcome cases)^A^	Number
	AUC, AUPRC, F1-score^A^	Values
	Accuracy, sensitivity (or recall), specificity, and positive predictive value (or precision) reported? ^A^	Yes or No
	MSE/MAE reported? ^A^	Yes or No
	ROC/PR curves presented? ^A^	Yes or No
	Calibration plot or metrics presented? ^AB^	Yes or No
8. External validation	Type of external validation^A^	Same or another department, center, or country
	Same items as internal validation	
9. Explainability	Global feature importance presented?*	Yes or No
	Single patient (local) feature importance presented?*	Yes or No
10. Final model availability	Is the final model directly available to apply to different data? ^A^	Yes or No
	Is the study code available to reproduce the methods?*	Yes or No

*Notes*: Data item sources indicated by A: CHARMS and B: TRIPOD; an asterisk (*) indicates data items added to the review protocol.

Abbreviations: BoW: Bag-of-Words; CE: Concept Extraction; WE: word embedding; DE: document embedding; TM: topic model; SS: summarizing score; LogR: logistic regression; LinR: linear regression; Cox: cox proportional hazards regression; NB: Naïve Bayes; RFTB: Random forests or other tree-based methods; GB: gradient boosting; SVM: support vector machines ; NN: neural networks; RNN: recurrent neural networks; CNN: convolutional neural networks; DNN: deep neural networks; AUC: area under the receiver operating characteristic curve; AUPRC: area under the precision-recall curve; MSE: mean squared error; MAE: mean absolute error; CHARMS: critical appraisal and data extraction for systematic reviews of prediction modeling studies; TRIPOD: transparent reporting of a multivariable prediction model for individual prognosis or diagnosis.

The input for text representation methods consisted of a list of both sparse and dense numeric vector representations. Sparse representations included Bag-of-Words, Term Frequency—Inverse Document Frequency (TFIDF), and clinical concept extraction. Dense vector representations included topic models, word and document embeddings, and summarizing scores, such as a sentiment score. Combinations of representations were possible. The machine learning methods were of varying complexity and interpretability, ranging from methods with relatively low complexity and high interpretability, such as linear or logistic regression, to increasingly more complex random forests, gradient boosting, support vector machines (*SVM*), and deep neural network methods.

Model explainability indicates whether a model is understandable to humans and whether the model’s behavior can be described in the entire feature space.^18^ However, determining the explainability of a model is not an objective or trivial task.^19^ Therefore, we assessed instead if a study paid attention to model explainability, regardless of whether the developed model could be considered explainable or not. This was assessed by the presentation of global feature importance, the feature importance over all predictions, or local feature importance, the contribution of features to a specific prediction.

If a study reported on multiple prediction problems, for example, a study reporting on both hospital readmission and in-hospital mortality prediction during intensive care unit (ICU) admission, the data items were extracted for each reported problem separately. The model and validation data items were only extracted for the—self-reported—best performing structured, text, and combined-data models in each problem. For data items with free text input, the results were manually categorized after data extraction to enable analysis.

We performed a meta-analysis on the reported model performance, comparing the structured, text, and combined-data models, for each prediction problem. The differences in the area under the receiver operating characteristic curve (AUC) were calculated for each reported feature set comparison: text and structured data (ΔAUC_TS_ = AUC_T_–AUC_S_), combined and structured data (ΔAUC_CS_ = AUC_C_–AUC_S_), and combined and text data (ΔAUC_CT_ = AUC_C_-AUC_T_). The AUC differences indicate the relative performance difference between the uses of the 3 feature sets within each prediction problem and are suitable to be compared across studies.

## RESULTS

### Search and data-extraction results

**Figure 2.**
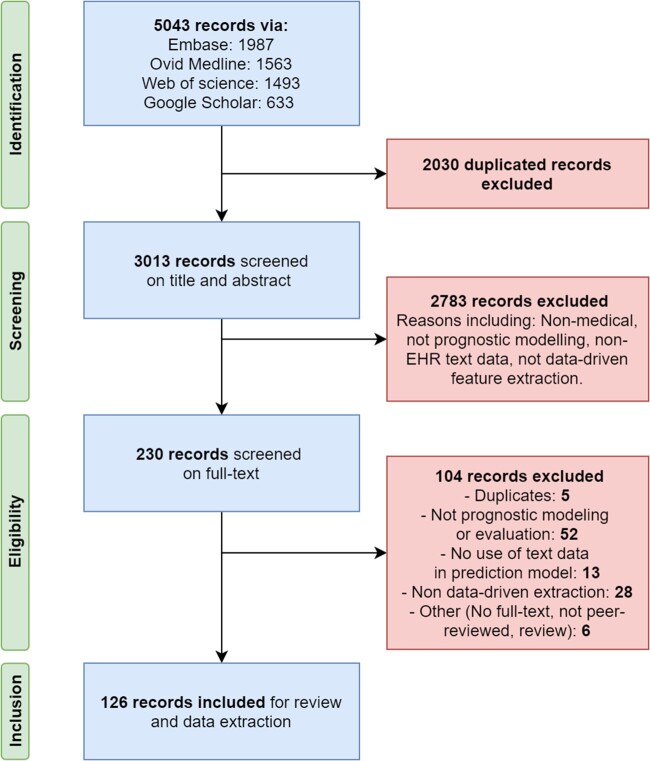
Preferred Reporting Items for Systematic Reviews and Meta-Analyses flow diagram with the search and screening results of the systematic review.

The literature search, performed in March 2021, resulted in a total of 5043 studies. The PRISMA flow diagram is presented in Figure 2. After deduplication, removing 2030 studies, a set of 3013 studies was screened on title and abstract. We excluded 2783 studies that violated one of the inclusion criteria. Full-text screening of the remaining 230 studies resulted in 126 relevant studies to be included in the review. The 104 studies that were excluded based on their full text consisted of 5 duplicate studies, 52 studies not performing prognostic modeling or a performance evaluation, 13 studies with no use of text data in the prediction model, 28 studies without data-driven information extraction, and 6 studies with other reasons for exclusion (no full-text available, not peer-reviewed, reviews). The study quality assessment, measured by TRIPOD adherence, resulted in an average score of 23.1 out of 30 (median = 24, min = 17, max = 28, *n* = 124) for the development only studies and 30.5 out of 36 for development and external validation studies (median = 30.5, min = 30, max = 31, *n* = 2). All included studies were considered to be of sufficient quality. The scoring table is available as Supplementary Material.

We extracted the different data items for each study and its reported prediction problems. Fourteen of the 126 studies reported multiple prediction problems, resulting in a total of 145 problems. A list of characteristics of the studies ordered by publication year is presented in Supplementary Table S2 and the extracted data items are available as Supplementary Material. The large majority of the reviewed studies (99, 79%) were published in the period from January 2018 to March 2021 (Table 3). No eligible studies were found in the period 2005–2011. The studies were published in a variety of journals and conference proceedings. The journals with the highest number of studies were the *Journal of Biomedical Informatics* (11, 9%), *PLoS One* (7, 6%), *BMC Medical Informatics and Decision Making* (6, 5%), *JMIR Medical Informatics* (6, 5%), and *The Journal of the American Medical Informatics Association* (6, 5%).

**Table 3.** Number of included studies by publication year

Year	2012	2013	2014	2015	2016	2017	2018	2019	2020	2021 (until March)
Number of included studies	4	0	5	4	9	5	19	30	41	9

Sixty-five of the 126 reviewed studies compared models that used structured data (S), text data (T), or a combination of structured and text data (C). A comparison between all 3 feature sets (S:T:C) was reported for 34 of the 145 prediction problems (23%). In 37 problems (26%) 2 feature sets were compared (S:T 6, 4%; S:C 26, 18%; T:C 5, 3%), and in 74 problems (51%) no comparison was made and the use of only 1 feature set was reported (T 48, 33%; C 26, 18%).

### Clinical settings and prediction problems

Most prediction problems focused on general hospital care settings (68, 47%), followed by intensive care (26, 18%), emergency care (20, 14%), surgical care (12, 8%), and psychiatric or mental health care (10, 7%). Only a few problems (5, 6%) were set in outpatient, or radiology settings. Almost half (68, 47%) of the problems used a local proprietary dataset, 18 problems (13%) used a collection of 2 or more local datasets, and 10 (7%) used registry, claims, or survey datasets. One-third of the prediction problems (48, 33%) were developed on a publicly available dataset. Specifically, 47 problems used the *Multiparameter Intelligent Monitoring in Intensive Care II* (MIMIC-II)^20^ database or the *Medical Information Mart for Intensive Care III* (MIMIC-III)^21^ database and one problem used the public dataset from the 2014 i2b2 Shared Task.^22^

**Figure 3.**
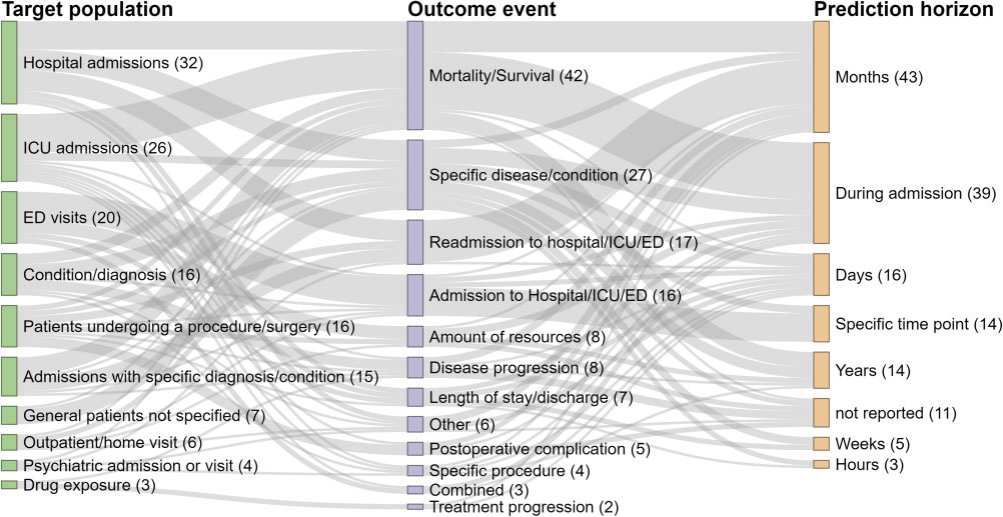
Sankey diagram of the different categories of target populations and clinical outcomes, and clinical outcomes and prediction horizons, ordered by size. The number in parentheses indicates the number of prediction problems with these categories and the width of the connection between 2 categories represents the number of prediction problems with this combination of categories.

Figure 3 visualizes the different categories of target populations, clinical outcomes, and prediction horizons that make up each prediction problem. The 3 largest target populations were patients with general hospital admissions (32, 22%), ICU admissions (26, 18%), and emergency department (ED) visits (20, 14%). The largest outcome events were mortality (42, 29%), diagnosis of a specific disease or condition (27, 19%), and hospital, ICU, or ED readmission (17, 12%). Most prediction problems had as prediction horizon a period of months (43, 30%) or the period during admission (39, 27%). There were 82 unique problems, combinations of a target population, outcome, and prediction horizon, of which 58 only occurred once. The prediction of mortality during admission in ICU patients occurred most often (10, 7%), followed by the prediction of admission to the hospital, including transfer to the ICU, at ED discharge for patients visiting the ED (9, 6%).

The observation window, in which the predictors are measured prior to the time of prediction, was not reported in 18 problems (12%). The most-reported observation windows, in all models, were the first 24 hours of a hospital or ICU admission or the first hour of the ED visit (50, 20%), during the entire admission or visit (36, 15%), and during triage (17, 10%). The relationship between the observation window and prediction horizon in text and combined-data models is visualized in Supplementary Figure S1. The most frequent combinations were an observation period during the first 24 hours of a hospital or ICU admission or the first hour of an ED visit followed by a prediction horizon during this admission or visit (24, 13%) and an observation period during the entire admission or visit followed by a prediction horizon in months (17, 9%).

**Figure 4.**
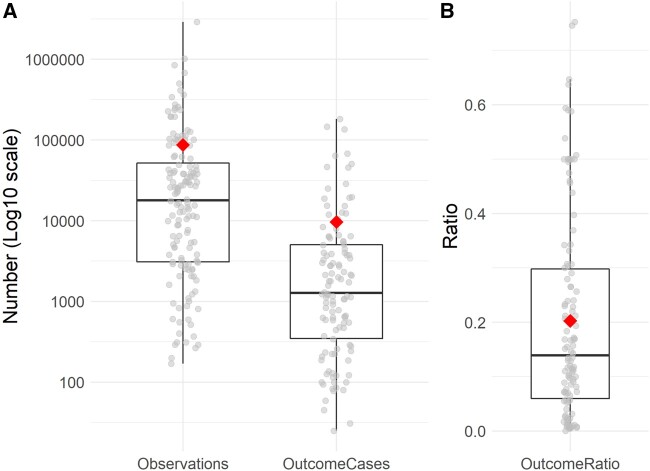
(A) Boxplots of the number of observations (left) and outcome cases (right) of 145 prediction problems. (B) Boxplot of the ratios between the number of observations and outcome cases. In both (A) and (B), the mean is indicated by the diamond and the points represent the underlying data.

The distributions of the number of observations and the number of observations with the outcome (outcome cases) are depicted in Figure 4A together with the distribution of their ratio in Figure 4B. The number of observations differed much between studies, from only a few hundred observations to a few million, with a mean of 87 016 and a median of 17 973 observations. Observations and outcome cases had an average ratio of 0.20 and a median of 0.14. In only 1 prediction problem, the number of observations was not reported and in 22 problems (16%) the number of outcome cases was missing.

### Preprocessing methods and text representations

Text preprocessing methods, applied before the text representation creation, were well-reported in 107 prediction problems (74%). The preprocessing of text commonly included methods such as sentence splitting, tokenization, lemmatization, the removal of stop words, punctuation, or numbers, abbreviation disambiguation, and the filtering of tokens based on frequencies. The text data was written in English for the majority of problems (115, 79%), followed by Chinese (8, 6%) and Portuguese (5, 3%). In total 12 different languages were reported. Various types of unstructured text were used, which we categorized into 17 categories, see Supplementary Figure S2. In 68 problems (47%) the type was unspecified. The most occurring types of unstructured text were nursing (19, 13%), physician (18, 12%), radiology (17, 12%), and triage notes (10, 7%), and surgery and (pre)operative reports (9, 6%).

Bag-of-Words and TFIDF text representations were used most often, in 67 of the 184 text and combined-data models (36%), followed by word embeddings (33, 18%) and concept extraction (24, 14%). In some cases, multiple representations were combined by concatenation (11, 6%) or dense representations were generated from extracted concepts (5, 3%). Dense representations had a median dimension of 200 features against 6985 features in sparse representations (Supplementary Figure S3). Preprocessing methods were more frequently reported together with the use of Bag-of-Words (34, 83%) and TFIDF (24, 92%) compared with concept extraction (14, 58%), document embeddings (11, 58%), and word embedding (25, 76%) methods, which often used out-of-the-box tools or software. *MetaMap*^23^ was the most common software used for extracting clinical concepts from text data, in 11 out of the 30 models using extracted concepts. Medical concepts from vocabularies in the Unified Medical Language System^24^ were extracted from the text data in 26 of 30 models. Five models made only use of the SNOMED Clinical Terms^25^ and for 4 models no ontology was reported.

### Machine learning methods

The most used methods for training text and combined-data models were logistic regression (49, 27%), recurrent neural networks (23, 13%), and random forest or other tree-based methods (19, 10%). For the structured-data models, logistic regression was also the most prevalent method (20, 30%), followed by gradient boosting (13, 20%) and recurrent neural networks (8, 12%). In most prediction problems (129, 89%), the prediction outcome was binary. Multi-class (10, 7%) and continuous (6, 4%) outcomes were used less often.

**Figure 5.**
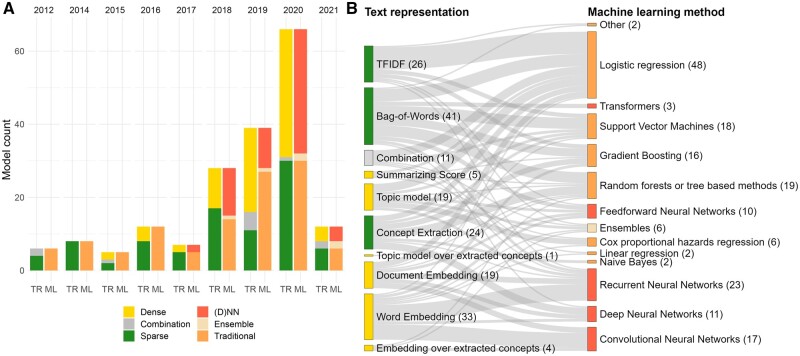
(A) The use of different text representations (TR) and machine learning (ML) methods in text-based or combined-data prediction models over time. No eligible studies in 2013. (D)NN are all feedforward and deep neural network-based methods. (B) The combinations of text representations (left) and machine-learning methods (right) in text-based or combined-data prediction models. The number in parentheses indicates the number of prediction problems with these categories and the width of the connection between 2 categories represents the number of prediction problems with this combination of categories. Both (A) and (B) share the same legend: the colors of the nodes indicate the types of text representations and machine learning methods.

Figure 5A depicts the use of both text representations (abstracted as dense, sparse, or combined representations) and machine learning methods in text and combined-data models over the years. A distinction was made between neural network-based methods, ensemble methods, and traditional machine learning methods not based on neural networks. It can be observed that until 2017, the use of sparse representations and traditional models was most common, but after 2018 the use of both dense text representations and neural networks took off. To understand their joined rise, we examined the relationship between the model’s machine learning method and its textual input representation in Figure 5B. It shows that the word and document embeddings served primarily as the input for deep learning methods, while the Bag-of-Words representations were commonly used by more traditional machine learning methods. The text representations and machine learning methods were significantly associated, *X*^2^ (4, *n* = 183) = 36.1, *P* < .001.

### Model performance evaluation and comparison

The internal validation model performance was reported using the AUC (or c-statistic/index) for the majority of prediction problems (121, 83%). For the other problems only metrics that are based on dichotomized outcomes, such as accuracy, sensitivity (or recall), specificity, and positive predictive value (or precision), were reported. The mean squared error or mean absolute error were reported for models predicting a continuous outcome (4, 3%). The F1-score was reported for 46 problems (31%) and the area under the precision-recall curve (AUPRC) for 20 (14%). The combined reporting of metrics is visualized in Supplementary Figure S4. A receiver operator curve or precision-recall curve was presented for 57 problems (39%), but for only 18 problems (12%) a calibration plot or calibration metric (such as the brier-score or calibration intercept and slope) was presented.

**Figure 6.**
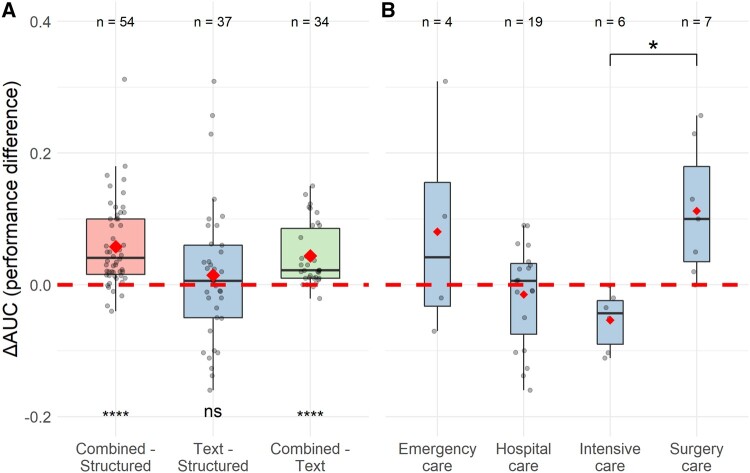
(A) Area under the receiver operating characteristic curve (AUC) difference distribution boxplots of the combined and structured-data models (ΔAUC Combined−Structured), the text and structured-data models (ΔAUC Text−Structured), and combined and text-based models (ΔAUC Combined−text). (B) Text and structured-data model AUC difference (ΔAUC Text−Structured) boxplots for 4 different clinical settings. In both (A) and (B), the means are indicated by a diamond, the points represent the underlying data, sample sizes are shown on top, and the dotted line indicates the AUC difference of zero. ns: not significant; *P < .05, ****P < .001.

Figure 6A depicts the distributions of AUC differences (ΔAUC) between the structured, text, and combined-data models within each prediction problem. The combined-data models had a visibly higher performance than the text or structured data models and the average AUC differences, for both ΔAUC_CS_ and ΔAUC_CT_, were significantly larger than zero, *t* (53)=6.76, *P* < .001 and *t* (33) = 5.49, *P* < .001 respectively. Text-based models did not perform significantly better or worse than the structured-data models. Their AUC difference, ΔAUC_TS_, showed a large variation across prediction problems. We investigated whether there was a relationship between these AUC differences and the clinical settings. Figure 6B shows how the text and structured-data model performance differences vary between 4 clinical settings: emergency, hospital, intensive, and surgical care. Psychiatric care is not presented as it only had 1 observation. Following a full pairwise comparison of the distributions, we found that the AUC difference means of the intensive and surgery care prediction problems were different *t* (8.55) = −3.95, *P* = .024 (Bonferroni adjusted). This implies that models using text data in the surgical care setting had on average a higher performance (compared with structured data models) than in the intensive care setting. This discrepancy may be caused by the use of different types of unstructured text in both care settings, see Supplementary Figure S2. Surgery care models incorporated primarily surgery and (pre)operative reports and radiology notes, while intensive care models relied on unspecified, nursing, physician, and radiology notes.

Notably, prediction models were externally validated in only 2 studies. Marafino et al^26^ externally validated their in-hospital mortality prediction model within 3 medical centers and Menger et al^27^ externally validated their in-hospital patient violence prediction model at 2 sites. Both studies reported a small to moderate decrease in external validation model performance, between 0.023 and 0.079 AUC difference.

### Model explainability and availability

The attention to model explainability was assessed by the presentation of global or local feature importance. Global feature importance was reported for 64 prediction problems (44%) and local feature importance was presented for 9 problems (6%). The final model was presented or made available in only 7 prediction problems (5%), whereas the code used for training the model was directly available online for 31 problems (21%).

## DISCUSSION

### Model performance

We found that in the 126 studies developing prognostic prediction models using unstructured text, published in the last 15 years, the performance of combined-data models on average outperformed the text and structured-data models. This demonstrates that the text data available in the EHR is a source of valuable information able to improve model performance in addition to structured data. Yan et al^14^ found comparable results in a review of 9 studies predicting sepsis. Although on average text-based models did not outperform structured-data models, we did see an interesting difference between clinical settings. In intensive care prediction problems, the structured-data models had a higher performance than the text-based models when compared with surgical care. This may be explained by the inherent differences in recording data between these clinical settings, as the intensive care is generally a structured-data rich setting, where the unstructured data had the form of physician and nursing notes, while in surgical care the information was contained in surgical and (pre)operative reports. Therefore, how clinical information is recorded may influence the performance of both text- and structured-based models.

### Clinical settings, datasets, and language

Hospital care (including intensive, surgical, and emergency care) was the most common clinical setting. While the combinations of different target populations, outcome events, and prediction horizons varied much between prediction problems, common themes could be observed, such as the prediction of mortality in the ICU or ED discharge disposition. Although almost half of the reviewed studies used a proprietary dataset, a third of the studies used a public dataset, specifically, the MIMIC-II^20^ or MIMIC-III^21^ dataset or the dataset from the 2014 i2b2 Shared Task.^22^ This shows that the public availability of datasets containing anonymized unstructured text and their use should be encouraged as they enable transparent and reproducible research on unstructured text and can serve as a benchmark for clinical NLP tasks. Almost 90% of the reviewed studies were performed on English unstructured text. This suggests that opportunities still exist for studying model development using text data in other languages.^28^^,^^29^

### Text processing and machine learning methods

The techniques used for preprocessing text and creating numeric text representations were generally well described. The impact of preprocessing methods on the model performance can be significant and those methods are therefore essential to report.^30^ The sparse Bag-of-Words and TFIDF representations and the dense word and document embeddings were most frequently used and we found an association between the types of text representation and machine learning methods. The neural network methods generally used a dense text representation, while regularized logistic regression methods, random forests, or SVMs largely took sparse representations as input.

### Model explainability and external validation

Less than half of the studies paid attention to model explainability, which may be considered rather limited given the importance of explainability and trustworthiness in clinical prediction models.^18^ When compared with logistic regression models, which are intrinsically interpretable, deep learning models need additional effort to be explained. Deep learning is well-suited for handling and combining structured and unstructured data,^31^ but the high complexity and dense input features impede direct explainability without posthoc explanation techniques.^18^ We suggest that future studies assess their model’s explainability and consider the use of either directly explainable modeling methods or posthoc explanation techniques.

Furthermore, only 2 studies presented external validation results and relatively few studies shared their trained model or code. Externally validating prediction models using text data might be challenging, due to the differences in (sub)language and EHR systems or the fear of sharing identifiable patient information captured by the model. However, assessing generalizability and external validity remains important in model development.^32^ Frameworks exist, such as the Observational Medical Outcomes Partnership Common Data Model (OMOP CDM),^33^ that deal with the lack of syntactic and semantic interoperability in health data. The OMOP CDM enables external validation by evaluating trained prediction models on other databases, only reporting back the aggregated and anonymized results.^1^^,^^3^ This allows research to meet the challenges of validating text-based models between databases using different languages. We suggest a future research focus on external validation and advocate the sharing of code or trained models for external validation by others. These steps will not only expand the model’s generalizability but will also promote the use of robust and trustworthy prediction models in clinical practice.^32^

### Strengths and limitations

There were likely some published studies eligible for inclusion that we did not find. For example, studies that incorporated text data in a prediction model but did not mention it in the title or abstract would have been missed. Nonetheless, the search query was designed to capture a wide variety of terms that would indicate the use of unstructured text. Furthermore, the level of granularity for predefined categories for text representations or machine learning methods was high. More granular categories on, for example, the different deep learning architectures could have been collected for more detailed and in-depth information. However, this would also have resulted in decreasing numbers per category, complicating interpretation. To the best of our knowledge, this is the first systematic review on the development of prognostic clinical prediction models using unstructured text. We performed a broad literature search over a long period of time, resulting in a large set of eligible studies in a wide variety of clinical settings, not focused on 1 specific prediction problem. The comparison of the relative performance between text, structured, and combined feature sets within each study allowed us to assess the value of text data in prediction model development. Finally, we made the extracted data available to provide transparency and reproducibility.

## CONCLUSION

In this systematic review, we found that the use of unstructured text in the development of prognostic prediction models was beneficial in most studies. Combining unstructured text with structured data in prediction model development generally improved model performance, while the performance of text-based models compared with structured-data models varied. Overall, unstructured text in EHR data should not be neglected, as it is a source of valuable information that can improve prediction model performance in addition to structured data. But the information available in both structured and unstructured data is likely dependent on the clinical setting and type of unstructured text. Models were generally developed in hospital care settings using a variety of text representations and machine learning methods and we found a relationship between the types of text representation and machine learning methods used. Furthermore, it is a cause for concern that only 2 studies externally validated their developed prediction models and that many studies had little attention for model explainability. Therefore, we suggest a focus on external validation and model explainability in future research. Additionally, we emphasize the importance of studying the use of text in non-English languages in prediction model development.

## FUNDING

This work has received support from the European Health Data & Evidence Network (EHDEN) project. EHDEN has received funding from the Innovative Medicines Initiative 2 Joint Undertaking (JU) under grant agreement No. 806968. The JU receives support from the European Union’s Horizon 2020 research and innovation program and EFPIA.

## AUTHOR CONTRIBUTIONS

TS designed the review protocol and performed the literature search. EF, SI, DJ, LJ, JK, AM, VP, AR, TS, RW, and CY performed the independent screening of studies. JK, EF, EM, and PR provided critical feedback, helped interpret the results and shape the research and analysis. TS performed the data extraction and analysis and wrote the article with input from all other authors.

## SUPPLEMENTARY MATERIAL

Supplementary material is available at *Journal of the American Medical Informatics Association* online.

## Supplementary Material

ocac058_Supplementary_Data
