# Supplementary material for: Use of unstructured text in prognostic clinical prediction models: a systematic review
Source: J Am Med Inform Assoc. 2022 Apr 27;29(7):1292–302. doi: 10.1093/jamia/ocac058 (PMC9196702; doi:10.1093/jamia/ocac058)
Supplement: ocac058_Supplementary_Data [file ocac058_supplementary_data.zip › Supplementary References.docx]

# REFERENCES

34. Halpern Y, Horng S, Nathanson LA. A comparison of dimensionality reduction techniques for unstructured clinical text. *ICML 2012 Workshop on Clinical Data Analysis* 2012.

35. Karnik S, Tan SL, Berg B, et al. Predicting atrial fibrillation and flutter using electronic health records. *Annu Int Conf IEEE Eng Med Biol Soc* 2012;2012:5562-65.

36. Lehman LW, Saeed M, Long W, et al. Risk stratification of ICU patients using topic models inferred from unstructured progress notes. *AMIA Annu Symp Proc* 2012;2012:505-11.

37. Li J, Guo L, Handly N, et al. Semantic-enhanced models to support timely admission prediction at emergency departments. *Netw Model Anal Health Inform Bioinform* 2012;1(4):161-72 doi: 10.1007/s13721-012-0014-6.

38. Huang SH, LePendu P, Iyer SV, et al. Toward personalizing treatment for depression: Predicting diagnosis and severity. *J Am Med Inform Assoc* 2014;21(6):1069-75 doi: 10.1136/amiajnl-2014-002733.

39. Huddar V, Rajan V, Bhattacharya S, et al. Predicting postoperative acute respiratory failure in critical care using nursing notes and physiological signals. *Annu Int Conf IEEE Eng Med Biol Soc* 2014;2014:2702-05 doi: 10.1109/EMBC.2014.6944180.

40. Kontio E, Airola A, Pahikkala T, et al. Predicting patient acuity from electronic patient records. *J Biomed Inform* 2014;51:35-40 doi: 10.1016/j.jbi.2014.04.001.

41. Poulin C, Shiner B, Thompson P, et al. Predicting the risk of suicide by analyzing the text of clinical notes. *PLoS One* 2014;9(1) doi: 10.1371/journal.pone.0085733.

42. Walsh C, Hripcsak G. The effects of data sources, cohort selection, and outcome definition on a predictive model of risk of thirty-day hospital readmissions. *J Biomed Inform* 2014 doi: 10.1016/j.jbi.2014.08.006.

43. Caballero K, Akella R. Dynamic Estimation of the Probability of Patient Readmission to the ICU using Electronic Medical Records. *AMIA Annu Symp Proc* 2015;2015:1831-40.

44. Marafino BJ, Boscardin WJ, Dudley RA. Efficient and sparse feature selection for biomedical text classification via the elastic net: Application to ICU risk stratification from nursing notes. *J Biomed Inform* 2015;54:114-20 doi: 10.1016/j.jbi.2015.02.003.

45. Perotte A, Ranganath R, Hirsch JS, et al. Risk prediction for chronic kidney disease progression using heterogeneous electronic health record data and time series analysis. *J Am Med Inform Assoc* 2015;22(4):872-80 doi: 10.1093/jamia/ocv024.

46. Roysden N, Wright A. Predicting Health Care Utilization After Behavioral Health Referral Using Natural Language Processing and Machine Learning. *AMIA Annu Symp Proc* 2015;2015:2063-72.

47. Cohen KB, Glass B, Greiner HM, et al. Methodological Issues in Predicting Pediatric Epilepsy Surgery Candidates Through Natural Language Processing and Machine Learning. *Biomed Inform Insights* 2016;8:11-8 doi: 10.4137/BII.S38308.

48. Hassanpour S, Langlotz CP. Predicting High Imaging Utilization Based on Initial Radiology Reports: A Feasibility Study of Machine Learning. *Acad Radiol* 2016;23(1):84-89 doi: 10.1016/j.acra.2015.09.014.

49. Hu D, Huang Z, Chan TM, et al. Utilizing Chinese admission records for MACE prediction of acute coronary syndrome. *Int J Environ Res Public Health* 2016;13(9) doi: 10.3390/ijerph13090912.

50. Luo YF, Rumshisky A. Interpretable Topic Features for Post-ICU Mortality Prediction. *AMIA Annu Symp Proc* 2016;2016:827-36.

51. McCoy TH, Castro VM, Roberson AM, et al. Improving prediction of suicide and accidental death after discharge from general hospitals with natural language processing. *JAMA Psychiatry* 2016;73(10):1064-71 doi: 10.1001/jamapsychiatry.2016.2172.

52. Miotto R, Li L, Kidd BA, et al. Deep Patient: An Unsupervised Representation to Predict the Future of Patients from the Electronic Health Records. *Sci Rep* 2016;6:26094 doi: 10.1038/srep26094.

53. Rumshisky A, Ghassemi M, Naumann T, et al. Predicting early psychiatric readmission with natural language processing of narrative discharge summaries. *Transl Psychiatry* 2016;6(10):e921 doi: 10.1038/tp.2015.182.

54. Soguero-Ruiz C, Hindberg K, Mora-Jiménez I, et al. Predicting colorectal surgical complications using heterogeneous clinical data and kernel methods. *J Biomed Inform* 2016;61:87-96 doi: 10.1016/j.jbi.2016.03.008.

55. Temple MW, Lehmann CU, Fabbri D. Natural Language Processing for Cohort Discovery in a Discharge Prediction Model for the Neonatal ICU. *Appl Clin Inform* 2016;7(1):101-15 doi: 10.4338/ACI-2015-09-RA-0114.

56. Buchan K, Filannino M, Uzuner Ö. Automatic prediction of coronary artery disease from clinical narratives. *J Biomed Inform* 2017;72:23-32 doi: 10.1016/j.jbi.2017.06.019.

57. Frost DW, Vembu S, Wang J, et al. Using the Electronic Medical Record to Identify Patients at High Risk for Frequent Emergency Department Visits and High System Costs. *Am J Med* 2017;130(5):601.e17-01.e22 doi: 10.1016/j.amjmed.2016.12.008.

58. Hong SN, Son HJ, Choi SK, et al. A prediction model for advanced colorectal neoplasia in an asymptomatic screening population. *PLoS One* 2017;12(8) doi: 10.1371/journal.pone.0181040.

59. Lucini FR, Fogliatto FS, da Silveira GJC, et al. Text mining approach to predict hospital admissions using early medical records from the emergency department. *Int J Med Inform* 2017;100:1-8 doi: 10.1016/j.ijmedinf.2017.01.001.

60. Zhang XY, Kim J, Patzer RE, et al. Prediction of Emergency Department Hospital Admission Based on Natural Language Processing and Neural Networks. *Methods Inf Med* 2017;56(5):377-89 doi: 10.3414/ME17-01-0024.

61. Adamou M, Antoniou G, Greasidou E, et al. Toward automatic risk assessment to support suicide prevention. *Crisis* 2018.

62. Bahl M, Barzilay R, Yedidia AB, et al. High-Risk Breast Lesions: A Machine Learning Model to Predict Pathologic Upgrade and Reduce Unnecessary Surgical Excision. *Radiology* 2018;286(3):810-18 doi: 10.1148/radiol.2017170549.

63. Banerjee I, Gensheimer MF, Wood DJ, et al. Probabilistic Prognostic Estimates of Survival in Metastatic Cancer Patients (PPES-Met) Utilizing Free-Text Clinical Narratives. *Sci Rep* 2018;8(1):10037 doi: 10.1038/s41598-018-27946-5.

64. Boag W, Doss D, Naumann T, et al. What's in a Note? Unpacking Predictive Value in Clinical Note Representations. *AMIA Jt Summits Transl Sci Proc* 2018;2017:26-34.

65. Coulet A, Shah NH, Wack M, et al. Predicting the need for a reduced drug dose, at first prescription. *Sci Rep* 2018;8(1):15558 doi: 10.1038/s41598-018-33980-0.

66. Gligorijevic D, Stojanovic J, Satz W, et al. Deep attention model for triage of emergency department patients. *Proc SIAM Int Conf Data Min* 2018.

67. Golas SB, Shibahara T. A machine learning model to predict the risk of 30-day readmissions in patients with heart failure: a retrospective analysis of electronic medical records data. *BMC Med Inform Decis Mak* 2018.

68. Huang YX, Lee J, Wang S, et al. Privacy-Preserving Predictive Modeling: Harmonization of Contextual Embeddings From Different Sources. *JMIR Med Inform* 2018;6(2):278-91 doi: 10.2196/medinform.9455.

69. Ian ERWS, Tran N, Dubin JA, et al. Sentiment in nursing notes as an indicator of out-of-hospital mortality in intensive care patients. *PLoS One* 2018;13(6) doi: 10.1371/journal.pone.0198687.

70. Krishnan GS, Kamath SS. A supervised learning approach for ICU mortality prediction based on unstructured electrocardiogram text reports. *Nat Lang Process Inf Syst* 2018.

71. Li Y, Yao L, Mao C, et al. Early Prediction of Acute Kidney Injury in Critical Care Setting Using Clinical Notes. *Proceedings (IEEE Int Conf Bioinformatics Biomed)* 2018;2018:683-86 doi: 10.1109/bibm.2018.8621574.

72. Menger V, Scheepers F, Spruit M. Comparing Deep Learning and Classical Machine Learning Approaches for Predicting Inpatient Violence Incidents from Clinical Text. *Applied Sciences* 2018;8(6) doi: 10.3390/app8060981.

73. Parreco J, Hidalgo A, Kozol R, et al. Predicting mortality in the surgical intensive care unit using artificial intelligence and natural language processing of physician documentation. *Am Surg* 2018;84(7):1190-94 doi: 10.1177/000313481808400736.

74. Rajkomar A, Oren E, Chen K, et al. Scalable and accurate deep learning with electronic health records. *NPJ Digit Med* 2018;1 doi: 10.1038/s41746-018-0029-1.

75. Sundararaman A, Ramanathan SV, Thati R. Novel Approach to Predict Hospital Readmissions Using Feature Selection from Unstructured Data with Class Imbalance. *Big Data Res* 2018;13:65-75 doi: 10.1016/j.bdr.2018.05.004.

76. Sushil M, Šuster S, Luyckx K, et al. Patient representation learning and interpretable evaluation using clinical notes. *J Biomed Inform* 2018;84:103-13 doi: 10.1016/j.jbi.2018.06.016.

77. Weissman GE, Hubbard RA, Ungar LH, et al. Inclusion of unstructured clinical text improves early prediction of death or prolonged ICU stay. *Crit Care Med* 2018;46(7):1125-32 doi: 10.1097/CCM.0000000000003148.

78. Yang Y, Wang X, Huang Y, et al. Ontology-based venous thromboembolism risk factors mining and model developing from medical records. *Proceedings (IEEE Int Conf Bioinformatics Biomed)* 2018:1669-72.

79. Afshar M, Dligach D, Sharma B, et al. Development and application of a high throughput natural language processing architecture to convert all clinical documents in a clinical data warehouse into standardized medical vocabularies. *J Am Med Inform Assoc* 2019;26(11):1364-69 doi: 10.1093/jamia/ocz068.

80. Akbilgic O, Homayouni R, Heinrich K, et al. Unstructured Text in EMR Improves Prediction of Death after Surgery in Children. *Informatics (MDPI)* 2019;6(1) doi: 10.3390/informatics6010004.

81. Alvarez-Mellado E, Holderness E, Miller N. Assessing the Efficacy of Clinical Sentiment Analysis and Topic Extraction in Psychiatric Readmission Risk Prediction. *EMNLP (2019)* 2019.

82. Apostolova E, Uppal A, Galarraga JE, et al. Towards Reliable ARDS Clinical Decision Support: ARDS Patient Analytics with Free-text and Structured EMR Data. *AMIA Annu Symp Proc* 2019;2019:228-37.

83. Beeksma M, Verberne S, van den Bosch A, et al. Predicting life expectancy with a long short-term memory recurrent neural network using electronic medical records. *BMC Med Inform Decis Mak* 2019;19(1):36 doi: 10.1186/s12911-019-0775-2.

84. Brown AD, Kachura JR. Natural Language Processing of Radiology Reports in Patients With Hepatocellular Carcinoma to Predict Radiology Resource Utilization. *J Am Coll Radiol* 2019;16(6):840-44 doi: 10.1016/j.jacr.2018.12.004.

85. Chen IY, Szolovits P, Ghassemi M. Can AI Help Reduce Disparities in General Medical and Mental Health Care? *AMA J Ethics* 2019;21(2):E167-E79 doi: 10.1001/amajethics.2019.167.

86. da Silva DA, ten Caten CS, dos Santos RP, et al. Predicting the occurrence of surgical site infections using text mining and machine learning. *PLoS One* 2019;14(12) doi: 10.1371/journal.pone.0226272.

87. Danielsen AA, Fenger MHJ, Østergaard SD, et al. Predicting mechanical restraint of psychiatric inpatients by applying machine learning on electronic health data. *Acta Psychiatr Scand* 2019;140(2):147-57 doi: 10.1111/acps.13061.

88. Danilov G, Kotik K, Shifrin M, et al. Prediction of Postoperative Hospital Stay with Deep Learning Based on 101 654 Operative Reports in Neurosurgery. *Stud Health Technol Inform* 2019;258:125-29.

89. Gong J, Bai X, Li DA, et al. Prognosis Analysis of Heart Failure Based on Recurrent Attention Model. *Ing Rech Biomed* 2020;41(2):71-79 doi: 10.1016/j.irbm.2019.08.002.

90. Khadanga S, Aggarwal K, Joty S. Using clinical notes with time series data for ICU management. *EMNLP (2019)* 2019.

91. Kongburan W, Chignell M, Charoenkitkarn N, et al. Enhancing Predictive Power of Cluster-Boosted Regression With Text-Based Indexing. *IEEE Access* 2019;7:43394-405 doi: 10.1109/ACCESS.2019.2908032.

92. Korach ZT, Cato KD, Collins SA, et al. Unsupervised Machine Learning of Topics Documented by Nurses about Hospitalized Patients Prior to a Rapid-Response Event. *Appl Clin Inform* 2019;10(5):952-63 doi: 10.1055/s-0039-3401814.

93. Krishnan GS. Evaluating the quality of word representation models for unstructured clinical text based ICU mortality prediction. *Proc ICDCN* 2019.

94. Liu R, Greenstein JL, Sarma SV, et al. Natural Language Processing of Clinical Notes for Improved Early Prediction of Septic Shock in the ICU. *Annu Int Conf IEEE Eng Med Biol Soc* 2019;2019:6103-08 doi: 10.1109/EMBC.2019.8857819.

95. Mahajan SM, Ghani R. Combining Structured and Unstructured Data for Predicting Risk of Readmission for Heart Failure Patients. *Stud Health Technol Inform* 2019;264:238-42 doi: 10.3233/SHTI190219.

96. Makino M, Yoshimoto R, Ono M, et al. Artificial intelligence predicts the progression of diabetic kidney disease using big data machine learning. *Sci Rep* 2019;9(1):11862 doi: 10.1038/s41598-019-48263-5.

97. Nakayama JY, Hertzberg V, Ho JC. Making sense of abbreviations in nursing notes: A case study on mortality prediction. *AMIA Jt Summits Transl Sci Proc* 2019;2019:275-84.

98. Payrovnaziri SN, Barrett LA, Bis D, et al. Enhancing Prediction Models for One-Year Mortality in Patients with Acute Myocardial Infarction and Post Myocardial Infarction Syndrome. *Stud Health Technol Inform* 2019;264:273-77 doi: 10.3233/SHTI190226.

99. Ross EG, Jung K, Dudley JT, et al. Predicting Future Cardiovascular Events in Patients with Peripheral Artery Disease Using Electronic Health Record Data. *Circ Cardiovasc Qual Outcomes* 2019;12(3) doi: 10.1161/CIRCOUTCOMES.118.004741.

100. Shin B, Hogan J, Adams AB, et al. Multimodal ensemble approach to incorporate various types of clinical notes for predicting readmission. *IEEE EMBS Int Conf Biomed Health Inform* 2019.

101. Si Y, Roberts K. Deep Patient Representation of Clinical Notes via Multi-Task Learning for Mortality Prediction. *AMIA Jt Summits Transl Sci Proc* 2019;2019:779-88.

102. Sterling NW, Patzer RE, Di MY, et al. Prediction of emergency department patient disposition based on natural language processing of triage notes. *Int J Med Inform* 2019;129:184-88 doi: 10.1016/j.ijmedinf.2019.06.008.

103. Sun M, Baron J, Dighe A, et al. Early Prediction of Acute Kidney Injury in Critical Care Setting Using Clinical Notes and Structured Multivariate Physiological Measurements. *Stud Health Technol Inform* 2019;264:368-72 doi: 10.3233/SHTI190245.

104. Wang LQ, Sha L, Lakin JR, et al. Development and Validation of a Deep Learning Algorithm for Mortality Prediction in Selecting Patients With Dementia for Earlier Palliative Care Interventions. *JAMA Netw Open* 2019;2(7) doi: 10.1001/jamanetworkopen.2019.6972.

105. Weissman GE, Ungar LH, Harhay MO, et al. Construct validity of six sentiment analysis methods in the text of encounter notes of patients with critical illness. *J Biomed Inform* 2019;89:114-21 doi: 10.1016/j.jbi.2018.12.001.

106. Yang YQ, Wang X, Huang Y, et al. Ontology-based venous thromboembolism risk assessment model developing from medical records. *BMC Med Inform Decis Mak* 2019;19 doi: 10.1186/s12911-019-0856-2.

107. Zhang X, Bellolio MF, Medrano-Gracia P, et al. Use of natural language processing to improve predictive models for imaging utilization in children presenting to the emergency department. *BMC Med Inform Decis Mak* 2019;19(1):287 doi: 10.1186/s12911-019-1006-6.

108. Bacchi S, Gluck S, Tan YR, et al. Prediction of general medical admission length of stay with natural language processing and deep learning: a pilot study. *Intern Emerg Med* 2020;15(6):989-95 doi: 10.1007/s11739-019-02265-3.

109. Barash Y, Soffer S, Grossman E, et al. Alerting on mortality among patients discharged from the emergency department: A machine learning model. *Postgrad Med J* 2020 doi: 10.1136/postgradmedj-2020-138899.

110. Barber EL, Garg R, Persenaire C, et al. Natural language processing with machine learning to predict outcomes after ovarian cancer surgery. *Gynecol Oncol* 2021;160(1):182-86 doi: 10.1016/j.ygyno.2020.10.004.

111. Baxter SL, Klie AR, Saseendrakumar BR, et al. Predicting Mortality in Critical Care Patients with Fungemia Using Structured and Unstructured Data. *Annu Int Conf IEEE Eng Med Biol Soc* 2020;2020:5459-63 doi: 10.1109/EMBC44109.2020.9175287.

112. Ben Miled Z, Haas K, Black CM, et al. Predicting dementia with routine care EMR data. *Artif Intell Med* 2020;102 doi: 10.1016/j.artmed.2019.101771.

113. Chen YW, Zhang LG, Zhang J, et al. Preoperative Risk Prediction of Heart Failure with Numerical and Textual Attributes. *Int J Innov Comp Inf Control* 2020;16(6):2035-46 doi: 10.24507/ijicic.16.06.2035.

114. Chen WJ, Lu ZJ, You LJ, et al. Artificial Intelligence-Based Multimodal Risk Assessment Model for Surgical Site Infection (AMRAMS): Development and Validation Study. *JMIR Med Inform* 2020;8(6) doi: 10.2196/18186.

115. Chen CH, Hsieh JG, Cheng SL, et al. Emergency department disposition prediction using a deep neural network with integrated clinical narratives and structured data. *Int J Med Inform* 2020;139 doi: 10.1016/j.ijmedinf.2020.104146.

116. Chen CH, Hsieh JG, Cheng SL, et al. Early short-term prediction of emergency department length of stay using natural language processing for low-acuity outpatients. *Am J Emerg Med* 2020;38(11):2368-73 doi: 10.1016/j.ajem.2020.03.019.

117. Danilov G, Kotik K, Shifrin M, et al. Predicting Postoperative Hospital Stay in Neurosurgery with Recurrent Neural Networks Based on Operative Reports. *Stud Health Technol Inform* 2020;270:382-86 doi: 10.3233/SHTI200187.

118. Fernandes M, Mendes R, Vieira SM, et al. Predicting intensive care unit admission among patients presenting to the emergency department using machine learning and natural language processing. *PLoS One* 2020;15(3) doi: 10.1371/journal.pone.0229331.

119. Fernandes M, Mendes R, Vieira SM, et al. Risk of mortality and cardiopulmonary arrest in critical patients presenting to the emergency department using machine learning and natural language processing. *PLoS One* 2020;15(4) doi: 10.1371/journal.pone.0230876.

120. Gensheimer MF, Aggarwal S, Benson KRK, et al. Automated model versus treating physician for predicting survival time of patients with metastatic cancer. *J Am Med Inform Assoc* 2020 doi: 10.1093/jamia/ocaa290.

121. Goodwin TR, Demner-Fushman D. A customizable deep learning model for nosocomial risk prediction from critical care notes with indirect supervision. *J Am Med Inform Assoc* 2020;27(4):567-76 doi: 10.1093/jamia/ocaa004.

122. Guo WP, Xu ZM, Ye XJ, et al. A Time-Critical Topic Model for Predicting the Survival Time of Sepsis Patients. *Sci Program* 2020;2020 doi: 10.1155/2020/8884539.

123. Hane CA, Nori VS, Crown WH, et al. Predicting Onset of Dementia Using Clinical Notes and Machine Learning: Case-Control Study. *JMIR Med Inform* 2020;8(6):e17819 doi: 10.2196/17819.

124. Hashir M, Sawhney R. Towards unstructured mortality prediction with free-text clinical notes. *J Biomed Inform* 2020;108 doi: 10.1016/j.jbi.2020.103489.

125. Heo TS, Kim YS, Choi JM, et al. Prediction of stroke outcome using natural language processing-based machine learning of radiology report of brain MRI. *J Pers Med* 2020;10(4):1-11 doi: 10.3390/jpm10040286.

126. Hsu CC, Karnwal S, Mullainathan S. Characterizing the Value of Information in Medical Notes. *EMNLP (2020)* 2020.

127. Izquierdo JL, Ancochea J, Savana C-RG, et al. Clinical Characteristics and Prognostic Factors for Intensive Care Unit Admission of Patients With COVID-19: Retrospective Study Using Machine Learning and Natural Language Processing. *J Med Internet Res* 2020;22(10):e21801 doi: 10.2196/21801.

128. Korach ZT, Yang J, Rossetti SC, et al. Mining clinical phrases from nursing notes to discover risk factors of patient deterioration. *Int J Med Inform* 2020;135 doi: 10.1016/j.ijmedinf.2019.104053.

129. Le S, Allen A, Calvert J, et al. Development and Validation of a Convolutional Neural Network Model for ICU Acute Kidney Injury Prediction. *Kidney Int Rep* 2020 doi: 10.1016/j.ekir.2021.02.031.

130. Lee D, Jiang X, Yu H. Harmonized representation learning on dynamic EHR graphs. *J Biomed Inform* 2020;106 doi: 10.1016/j.jbi.2020.103426.

131. Levis M, Leonard Westgate C, Gui J, et al. Natural language processing of clinical mental health notes may add predictive value to existing suicide risk models. *Psychol Med* 2020:1-10 doi: 10.1017/S0033291720000173.

132. Li Y, Nair P, Lu XH, et al. Inferring multimodal latent topics from electronic health records. *Nat Commun* 2020;11(1) doi: 10.1038/s41467-020-16378-3.

133. Meng Y, Speier WF, Ong M, et al. HCET: Hierarchical Clinical Embedding with Topic Modeling on Electronic Health Record for Predicting Depression. *IEEE J Biomed Health Inform* 2020;PP doi: 10.1109/JBHI.2020.3004072.

134. Mohammadi R, Jain S, Namin AT, et al. Predicting Unplanned Readmissions Following a Hip or Knee Arthroplasty: Retrospective Observational Study. *JMIR Med Inform* 2020;8(11):e19761 doi: 10.2196/19761.

135. Mugisha C, Paik I. Pneumonia Outcome Prediction Using Structured And Unstructured Data From EHR. *Proceedings (IEEE Int Conf Bioinformatics Biomed)* 2020:2640-46.

136. Nakatani H, Nakao M, Uchiyama H, et al. Predicting Inpatient Falls Using Natural Language Processing of Nursing Records Obtained From Japanese Electronic Medical Records: Case-Control Study. *JMIR Med Inform* 2020;8(4):e16970 doi: 10.2196/16970.

137. Obeid JS, Dahne J, Christensen S, et al. Identifying and Predicting Intentional Self-Harm in Electronic Health Record Clinical Notes: Deep Learning Approach. *JMIR Med Inform* 2020;8(7):e17784 doi: 10.2196/17784.

138. Roquette BP, Nagano H, Marujo EC, et al. Prediction of admission in pediatric emergency department with deep neural networks and triage textual data. *Neural Netw* 2020;126:170-77 doi: 10.1016/j.neunet.2020.03.012.

139. Shukla SN, Marlin BM. Integrating Physiological Time Series and Clinical Notes with Deep Learning for Improved ICU Mortality Prediction. *Proc ACM Conf Health Inference Learn (2020)* 2020.

140. Sterckx L, Vandewiele G, Dehaene I, et al. Clinical information extraction for preterm birth risk prediction. *J Biomed Inform* 2020;110 doi: 10.1016/j.jbi.2020.103544.

141. Sterling NW, Brann F, Patzer RE, et al. Prediction of emergency department resource requirements during triage: An application of current natural language processing techniques. *J Am Coll Emerg Physicians Open* 2020;1(6):1676-83 doi: 10.1002/emp2.12253.

142. Tahayori B, Chini-Foroush N, Akhlaghi H. Advanced natural language processing technique to predict patient disposition based on emergency triage notes. *Emerg Med Australas* 2020 doi: 10.1111/1742-6723.13656.

143. Topaz M, Woo K, Ryvicker M, et al. Home Healthcare Clinical Notes Predict Patient Hospitalization and Emergency Department Visits. *Nurs Res* 2020;69(6):448-54 doi: 10.1097/NNR.0000000000000470.

144. Wang HY, Li YK, Khan SA, et al. Prediction of breast cancer distant recurrence using natural language processing and knowledge-guided convolutional neural network. *Artif Intell Med* 2020;110 doi: 10.1016/j.artmed.2020.101977.

145. Weegar R, Sundström K. Using machine learning for predicting cervical cancer from Swedish electronic health records by mining hierarchical representations. *PLoS One* 2020;15(8 August 2020) doi: 10.1371/journal.pone.0237911.

146. Xu L, Hogan J, Patzer RE, et al. Noise Pollution in Hospital Readmission Prediction: Long Document Classification with Reinforcement Learning. *2020 BioNLP ACL Workshop on Biomedical Natural Language Processing* 2020.

147. Ye JC, Yao L, Shen JH, et al. Predicting mortality in critically ill patients with diabetes using machine learning and clinical notes. *BMC Med Inform Decis Mak* 2020;20 doi: 10.1186/s12911-020-01318-4.

148. Zhang DD, Yin CC, Zeng JC, et al. Combining structured and unstructured data for predictive models: a deep learning approach. *BMC Med Inform Decis Mak* 2020;20(1) doi: 10.1186/s12911-020-01297-6.

149. Boag W, Kovaleva O, McCoy TH, et al. Hard for humans, hard for machines: predicting readmission after psychiatric hospitalization using narrative notes. *Transl Psychiatry* 2021;11(1) doi: 10.1038/s41398-020-01104-w.

150. Chen YP, Lo YH, Lai F, et al. Disease concept-embedding based on the self-supervised method for medical information extraction from electronic health records and disease retrieval: Algorithm development and validation study. *J Med Internet Res* 2021;23(1) doi: 10.2196/25113.

151. Goh KH, Wang L, Yeow AYK, et al. Artificial intelligence in sepsis early prediction and diagnosis using unstructured data in healthcare. *Nat Commun* 2021;12(1) doi: 10.1038/s41467-021-20910-4.

152. Klang E, Kummer BR, Dangayach NS, et al. Predicting adult neuroscience intensive care unit admission from emergency department triage using a retrospective, tabular-free text machine learning approach. *Sci Rep* 2021;11(1):1381 doi: 10.1038/s41598-021-80985-3.

153. Muhlestein WE, Monsour MA, Friedman GN, et al. Predicting Discharge Disposition Following Meningioma Resection Using a Multi-Institutional Natural Language Processing Model. *Neurosurgery* 2021 doi: 10.1093/neuros/nyaa585.

154. Oliwa T, Furner B, Schmitt J, et al. Development of a predictive model for retention in HIV care using natural language processing of clinical notes. *J Am Med Inform Assoc* 2021;28(1):104-12 doi: 10.1093/jamia/ocaa220.

155. Ribelles N, Jerez JM, Rodriguez-Brazzarola P, et al. Machine learning and natural language processing (NLP) approach to predict early progression to first-line treatment in real-world hormone receptor-positive (HR+)/HER2-negative advanced breast cancer patients. *Eur J Cancer* 2021;144:224-31 doi: 10.1016/j.ejca.2020.11.030.

156. Tang C, Plasek JM, Shi X, et al. Estimating Time to Progression of Chronic Obstructive Pulmonary Disease with Tolerance. *IEEE J Biomed Health Inform* 2021;25(1):175-80 doi: 10.1109/JBHI.2020.2992259.

157. Yang HY, Kuang L, Xia FQ. Multimodal temporal-clinical note network for mortality prediction. *J Biomed Semant* 2021;12(1) doi: 10.1186/s13326-021-00235-3.
