# Supplementary material for: Use of unstructured text in prognostic clinical prediction models: a systematic review
Source: J Am Med Inform Assoc. 2022 Apr 27;29(7):1292–302. doi: 10.1093/jamia/ocac058 (PMC9196702; doi:10.1093/jamia/ocac058)
Supplement: ocac058_Supplementary_Data [file ocac058_supplementary_data.zip › SystematicReview_Seinen_SupplementaryMaterial_revision.docx]

# SUPPLEMENTARY MATERIAL

## **Supplementary tables**

*Table S1. Search strategy*

| **Clause** | **Field** | **Search terms** |
| --- | --- | --- |
| 1 | Topic | 'prediction model' OR 'prediction' |
|  | Title, abstract, keywords | ((predict* OR prognost*) NEAR/3 (model* OR risk* OR rule*)) OR  ((early) NEAR/3 (predict* OR ident* OR risk*)) |
|  | Title | predict* OR prognost* OR  ((risk*) NEAR/6 (model* OR estimat* OR adjust* OR assess* OR identif* OR validat* OR forecast* OR scor* OR factor*)) |
| 2 | Topic | 'medical record'(explode) |
|  | Title, abstract, keywords | EHR OR EHRs OR EMR OR EMRs OR clinical* OR nursing OR nurse* OR physician* OR doctor* OR medical* OR healthcare* OR health-care OR patient* OR  ((electronic* OR medical) NEAR/3 (record* OR data*)) OR  ((patient*) NEAR/3 (record* OR histor* OR data*)) |
| 3 | Topic | 'natural language processing' OR 'text mining' |
|  | Title, abstract, keywords | NLP OR mine OR text_mining OR  ((data*) NEAR/4 (unstruct* or raw)) OR  ((natural) NEAR/3 (language* OR text*)) OR  ((unstruct* OR free OR form OR raw OR embedding) NEAR/4 (text OR textual* OR texts OR note OR notes OR phrase*)) OR  ((clinical* OR nursing OR nurse* OR doctor* OR medical* OR healthcare* OR health-care OR feature* OR patient*) NEAR/4 (text OR textual* OR texts OR note OR notes OR phrase*)) OR  ((text OR word* OR textual* OR texts OR note OR notes OR phrase* OR unstruct* OR linguist* OR semantic*)NEAR/4 (feature* OR predictor* OR covariate*)) OR  (topic near/2 model*) |
| 4 | Publication year | [2005-2020] |
|  | Limit | [English] |
|  | Limit | NOT [conference abstract] |

*Table S2. Characteristics of studies included in the review (n=126)*

| **Study** | **Publication**  **year** | **Country of dataset** | **Clinical care settings** | **Number of Prediction Problems** | **Feature Sets**  **compared** |
| --- | --- | --- | --- | --- | --- |
| Halpern [34] | 2012 | USA | Emergency care | 1 | T |
| Karnik [35] | 2012 | USA | Hospital care | 1 | T:S:C |
| Lehman [36] | 2012 | USA | Hospital care | 1 | T:S:C |
| Li [37] | 2012 | USA | Emergency care | 1 | C |
| Huang [38] | 2014 | USA | Psychiatric or mental health care | 2 | C |
| Huddar [39] | 2014 | USA | Intensive care | 1 | T |
| Kontio [40] | 2014 | Finland | Hospital care | 1 | T:S:C |
| Poulin [41] | 2014 | USA | Psychiatric or mental health care | 1 | T |
| Walsh [42] | 2014 | USA | Hospital care | 1 | T:S:C |
| Caballero [43] | 2015 | USA | Hospital care | 1 | S:C |
| Marafino [44] | 2015 | USA | Intensive care | 1 | T |
| Perotte [45] | 2015 | USA | Hospital care | 1 | T:S:C |
| Roysden [46] | 2015 | USA | Hospital care | 1 | C |
| Cohen [47] | 2016 | USA | Hospital care | 1 | T:C |
| Hassanpour [48] | 2016 | USA | Radiology | 1 | T |
| Hu [49] | 2016 | China | Hospital care | 1 | T |
| Luo [50] | 2016 | USA | Intensive care | 1 | S:C |
| McCoy [51] | 2016 | USA | Hospital care | 1 | S:C |
| Miotto [52] | 2016 | USA | Hospital care | 1 | C |
| Rumshisky [53] | 2016 | USA | Hospital care | 1 | S:C |
| Soguero-Ruiz [54] | 2016 | Norway | Hospital care | 1 | T:S:C |
| Temple [55] | 2016 | USA | Intensive care | 1 | T:S:C |
| Buchan [56] | 2017 | USA | Hospital care | 1 | T |
| Frost [57] | 2017 | Canada | Emergency care | 2 | T |
| Hong [58] | 2017 | South Korea | Hospital care | 1 | C |
| Lucini [59] | 2017 | Brazil | Emergency care | 1 | T |
| Zhang [60] | 2017 | USA | Emergency care | 1 | T:S:C |
| Adamou [61] | 2018 | UK | Psychiatric or mental health care | 1 | S:C |
| Bahl [62] | 2018 | USA | Hospital care | 1 | C |
| Banerjee [63] | 2018 | USA | Hospital care | 1 | T |
| Boag [64] | 2018 | USA | Intensive care | 4 | T |
| Coulet [65] | 2018 | USA | Hospital care | 1 | C |
| Gligorijevic [66] | 2018 | USA | Emergency care | 1 | T:S:C |
| Golas [67] | 2018 | USA | Hospital care | 1 | C |
| Huang [68] | 2018 | USA | Hospital care | 1 | C |
| Ian [69] | 2018 | USA | Intensive care | 1 | S:C |
| Krishnan [70] | 2018 | USA | Intensive care | 1 | T |
| Li [71] | 2018 | USA | Hospital care | 1 | T |
| Marafino [26] | 2018 | USA | Intensive care | 1 | S:C |
| Menger [72] | 2018 | Netherlands | Psychiatric or mental health care | 1 | T |
| Parreco [73] | 2018 | USA | Intensive care | 1 | T:S:C |
| Rajkomar [74] | 2018 | USA | Hospital care | 3 | C |
| Sundararaman [75] | 2018 | USA | Hospital care | 1 | T:S:C |
| Sushil [76] | 2018 | USA | Intensive care | 2 | T |
| Weissman [77] | 2018 | USA | Intensive care | 1 | S:C |
| Yang [78] | 2018 | China | Hospital care | 1 | T |
| Afshar [79] | 2019 | USA | Hospital care | 1 | T |
| Akbilgic [80] | 2019 | USA | Surgery care | 1 | T:S:C |
| Alvarez-Mellado [81] | 2019 | USA | Psychiatric or mental health care | 1 | S:C |
| Apostolova [82] | 2019 | USA | Hospital care | 1 | S:C |
| Beeksma [83] | 2019 | Netherlands | Outpatient care | 1 | S:C |
| Brown [84] | 2019 | USA | Surgery care | 1 | T |
| Chen [85] | 2019 | USA | Intensive care | 1 | T:C |
| da Silva [86] | 2019 | Brazil | Surgery care | 1 | T |
| Danielsen [87] | 2019 | Denmark | Psychiatric or mental health care | 1 | C |
| Danilov [88] | 2019 | Russia | Surgery care | 1 | T |
| Gong [89] | 2019 | China | Hospital care | 1 | C |
| Khadanga [90] | 2019 | USA | Intensive care | 2 | T:S:C |
| Kongburan [91] | 2019 | USA | Intensive care | 1 | S:C |
| Korach [92] | 2019 | USA | Hospital care | 1 | T:S:C |
| Krishnan [93] | 2019 | USA | Intensive care | 1 | T |
| Liu [94] | 2019 | USA | Intensive care | 1 | S:C |
| Mahajan [95] | 2019 | USA | Hospital care | 1 | T:S:C |
| Makino [96] | 2019 | Japan | Hospital care | 1 | S:C |
| Menger [27] | 2019 | Netherlands | Psychiatric or mental health care | 1 | T |
| Nakayama [97] | 2019 | USA | Hospital care | 1 | S:C |
| Payrovnaziri [98] | 2019 | USA | Hospital care | 1 | S:C |
| Ross [99] | 2019 | USA | Hospital care | 1 | S:C |
| Shin [100] | 2019 | USA | Hospital care | 1 | C |
| Si [101] | 2019 | USA | Hospital care | 1 | T |
| Sterling [102] | 2019 | USA | Emergency care | 1 | T |
| Sun [103] | 2019 | USA | Intensive care | 1 | T:S:C |
| Wang [104] | 2019 | USA | Hospital care | 1 | C |
| Weissman [105] | 2019 | USA | Hospital care | 1 | T |
| Yang [106] | 2019 | China | Hospital care | 1 | T |
| Zhang [107] | 2019 | USA | Emergency care | 1 | T:S:C |
| Bacchi [108] | 2020 | Australia | Emergency care | 2 | T |
| Barash [109] | 2020 | Israel | Intensive care | 1 | T:S:C |
| Barber [110] | 2020 | USA | Surgery care | 2 | T:S:C |
| Baxter [111] | 2020 | USA | Hospital care | 1 | T:S |
| Ben Miled [112] | 2020 | USA | unclear | 1 | T:C |
| Chen [113] | 2020 | China | Surgery care | 1 | S:C |
| Chen [114] | 2020 | China | Surgery care | 1 | T:S:C |
| Chen [115] | 2020 | Taiwan | Emergency care | 1 | S:C |
| Chen [116] | 2020 | Taiwan | Emergency care | 1 | S:C |
| Danilov [117] | 2020 | Russia | Surgery care | 1 | T |
| Fernandes [118] | 2020 | Portugal, USA | Emergency care | 2 | C |
| Fernandes [119] | 2020 | Portugal | Emergency care | 1 | S:C |
| Gensheimer [120] | 2020 | USA | Hospital care | 1 | C |
| Goodwin [121] | 2020 | USA | Hospital care | 3 | T |
| Guo [122] | 2020 | USA | Hospital care | 1 | C |
| Hane [123] | 2020 | USA | Claims | 1 | S:C |
| Hashir [124] | 2020 | USA | Hospital care | 1 | T:S:C |
| Heo [125] | 2020 | South-Korea | Hospital care | 1 | T |
| Hsu [126] | 2020 | USA | Hospital care | 2 | T:S:C |
| Izquierdo [127] | 2020 | Spain | Hospital care | 1 | T |
| Korach [128] | 2020 | USA | Hospital care | 1 | T |
| Le [129] | 2020 | USA | Intensive care | 1 | S:C |
| Lee [130] | 2020 | USA | Outpatient care | 2 | C |
| Levis [131] | 2020 | USA | Psychiatric or mental health care | 1 | T |
| Li [132] | 2020 | USA | Intensive care | 1 | C |
| Meng [133] | 2020 | not reported | unclear | 1 | S:C |
| Mohammadi [134] | 2020 | USA | Surgery care | 2 | T:S |
| Mugisha [135] | 2020 | USA | Intensive care | 1 | T:S:C |
| Nakatani [136] | 2020 | Japan | Hospital care | 1 | T |
| Obeid [137] | 2020 | USA | Hospital care | 1 | T |
| Roquette [138] | 2020 | Brazil | Hospital care | 1 | S:C |
| Shukla [139] | 2020 | USA | Hospital care | 1 | T:S:C |
| Sterckx [140] | 2020 | Belgium | Hospital care | 1 | T:S:C |
| Sterling [141] | 2020 | USA | Emergency care | 1 | C |
| Tahayori [142] | 2020 | Australia | Emergency care | 1 | T |
| Topaz [143] | 2020 | USA | Outpatient care | 1 | T |
| Wang [144] | 2020 | USA | Hospital care | 1 | T:C |
| Weegar [145] | 2020 | Sweden | Hospital care | 1 | T:S:C |
| Xu [146] | 2020 | USA | Hospital care | 1 | T |
| Ye [147] | 2020 | USA | Hospital care | 1 | T:S |
| Zhang [148] | 2020 | USA | Hospital care | 3 | T:S:C |
| Boag [149] | 2021 | USA | Psychiatric or mental health care | 1 | T:S:C |
| Chen [150] | 2021 | Taiwan | Emergency care | 1 | C |
| Goh [151] | 2021 | Singapore | Hospital care | 1 | S:C |
| Klang [152] | 2021 | USA | Emergency care | 1 | T:S:C |
| Muhlestein [153] | 2021 | USA | Surgery care | 1 | T:S |
| Oliwa [154] | 2021 | USA | Outpatient care | 1 | T |
| Ribelles [155] | 2021 | Spain | Hospital care | 1 | T:S |
| Tang [156] | 2021 | USA | Hospital care | 1 | T |
| Yang [157] | 2021 | USA | Hospital care | 1 | T:C |

## **Supplementary figures**

*Figure S1. The combinations of observation windows and prediction horizons over all text and combined-data models.*

*Figure S2. The usage of the different types of notes in four clinical settings.*

*Figure S3. The number of predictors in combined, dense, or sparse text representations. The mean is indicated by the red diamond and the points represent the underlying data.*

*Figure S4. Euler diagram depicting the combined reporting of different performance metrics in the 145 prediction problems. AUC: Area under the receiver operator curve, AUPRC: area under the precision-recall curve, MAE: Mean absolute error, MSE: mean squared error, F1: F1 score, Acc/Prec/Rec: reporting of accuracy, sensitivity (or recall), specificity, or positive predictive value (or precision).*

## **Supplementary data**

See files:

SystematicReview_Seinen_SupplementaryData_TripodAdherence.csv

SystematicReview_Seinen_SupplementaryData_DataExtraction.csv
