# Supplementary figures and images for: Use of unstructured text in prognostic clinical prediction models: a systematic review
Source: J Am Med Inform Assoc. 2022 Apr 27;29(7):1292–302. doi: 10.1093/jamia/ocac058 (PMC9196702; doi:10.1093/jamia/ocac058)

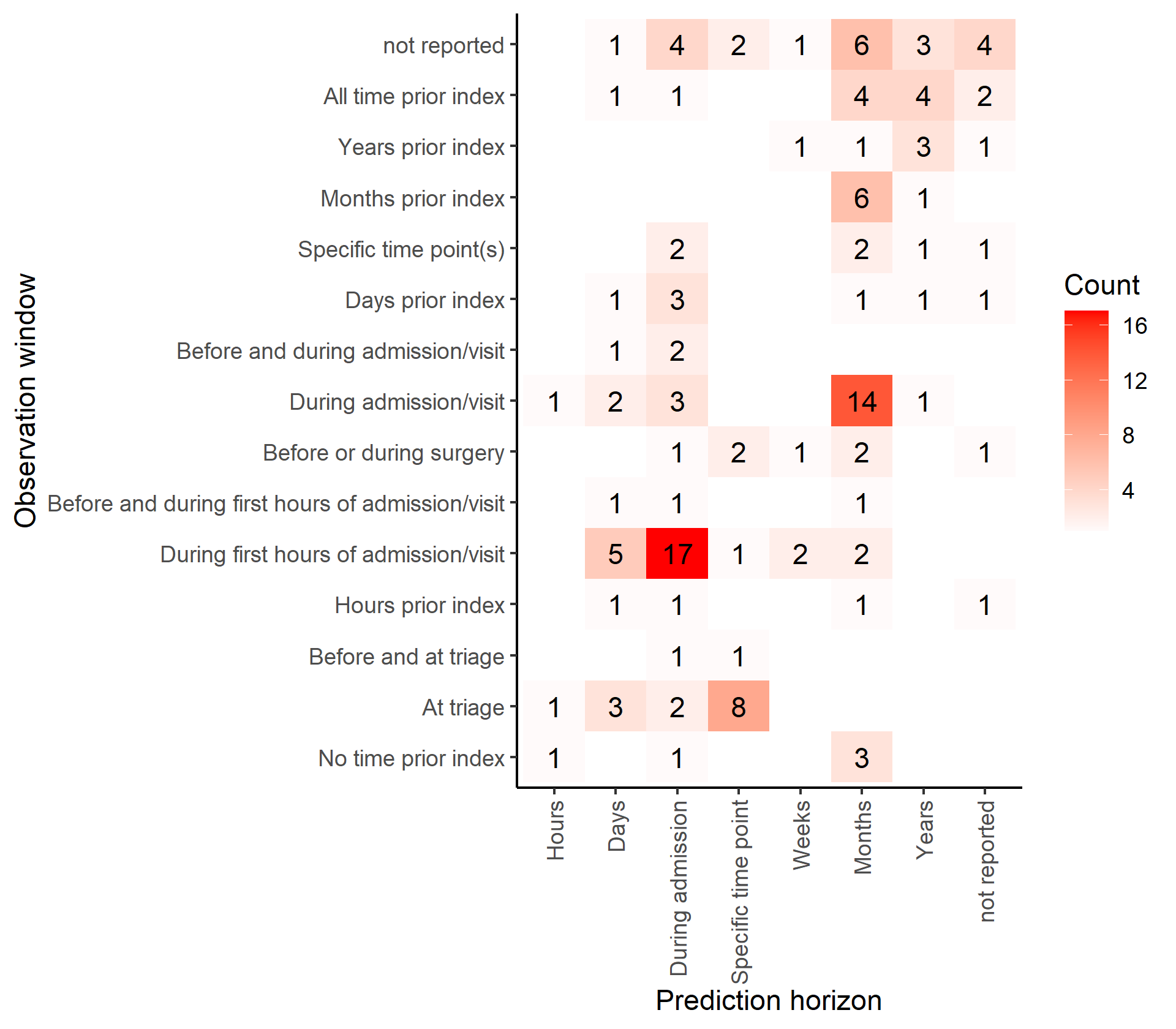

Supplement: ocac058_Supplementary_Data [file ocac058_supplementary_data.zip › FigureS1.png]

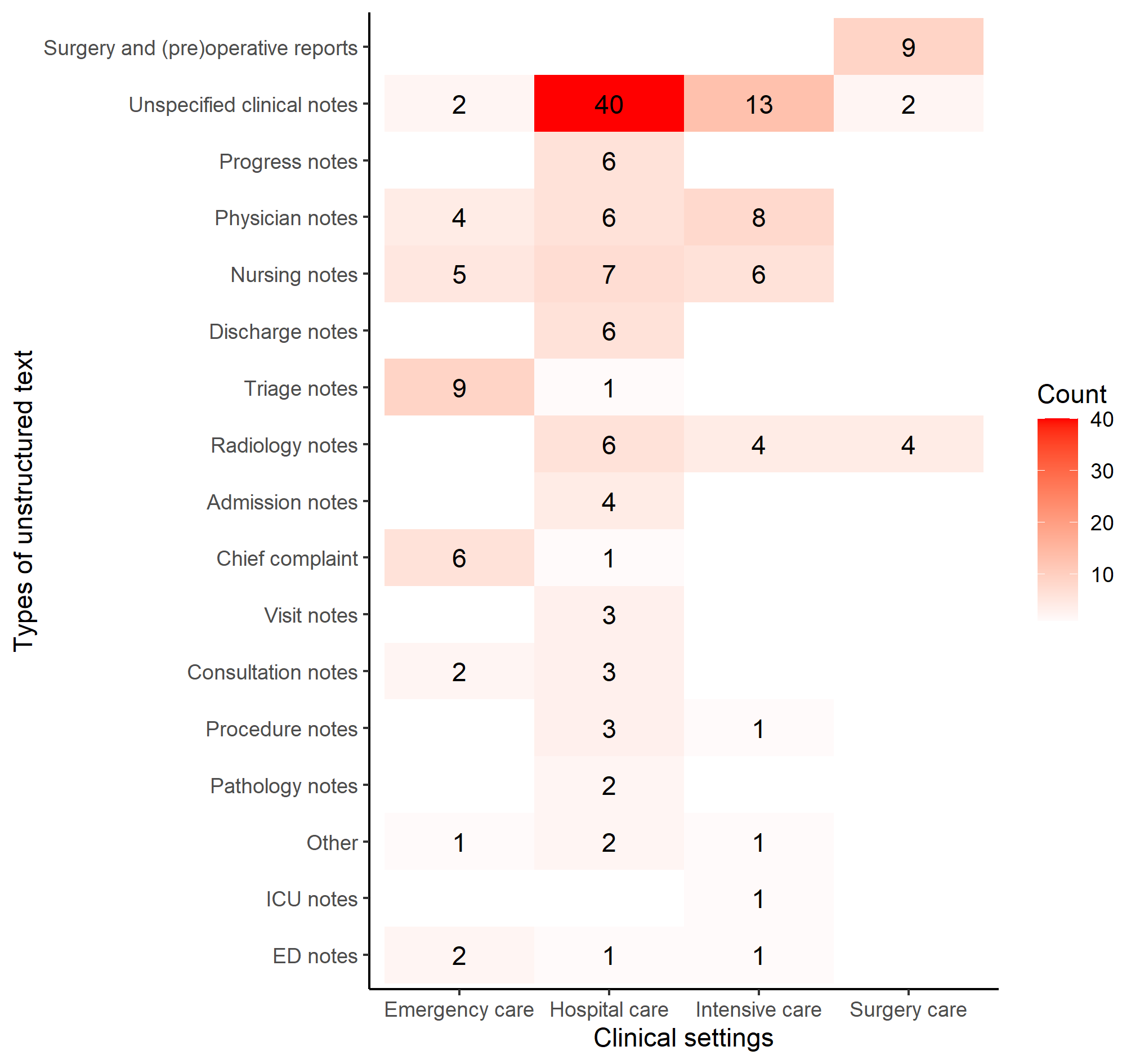

Supplement: ocac058_Supplementary_Data [file ocac058_supplementary_data.zip › FigureS2.png]

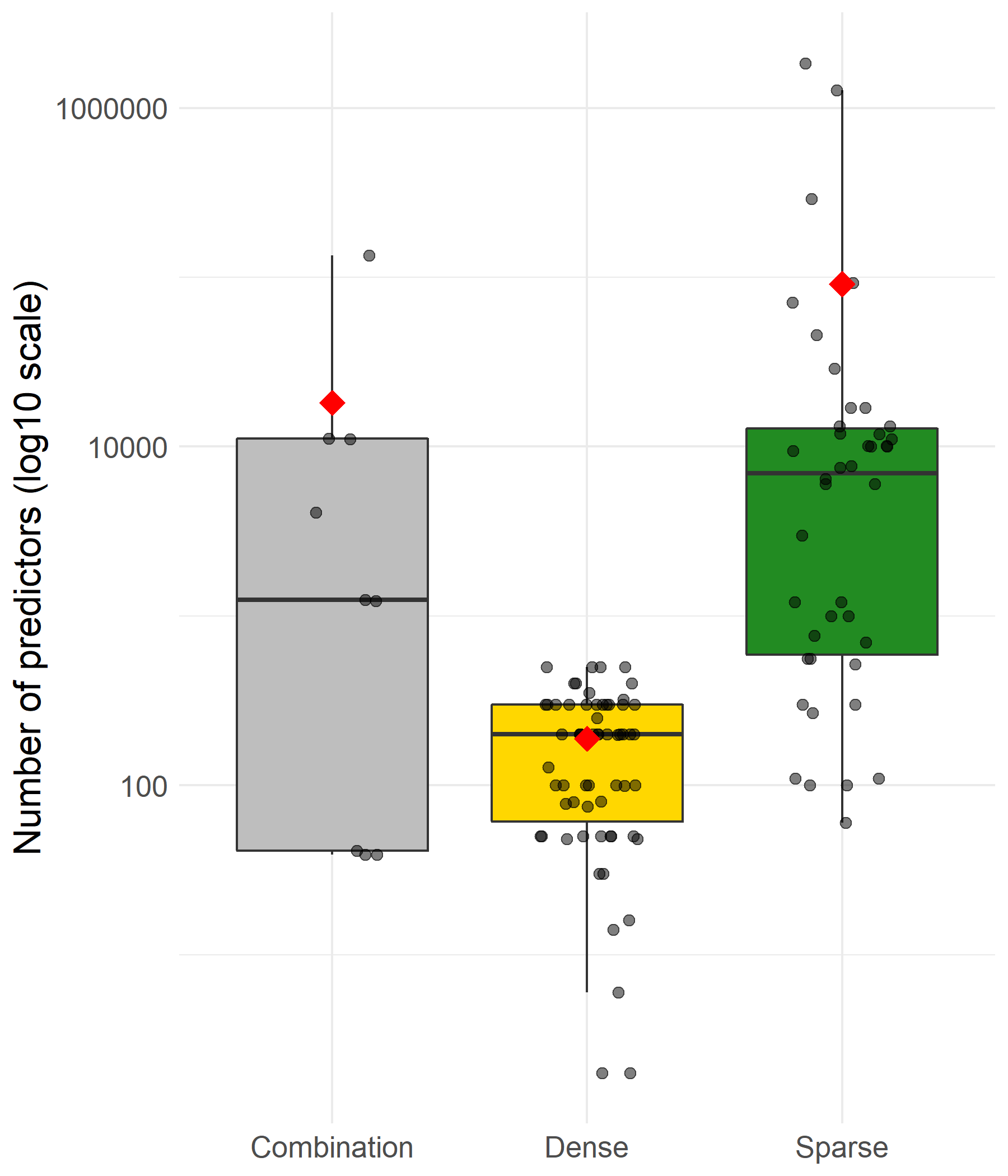

Supplement: ocac058_Supplementary_Data [file ocac058_supplementary_data.zip › FigureS3.tiff]

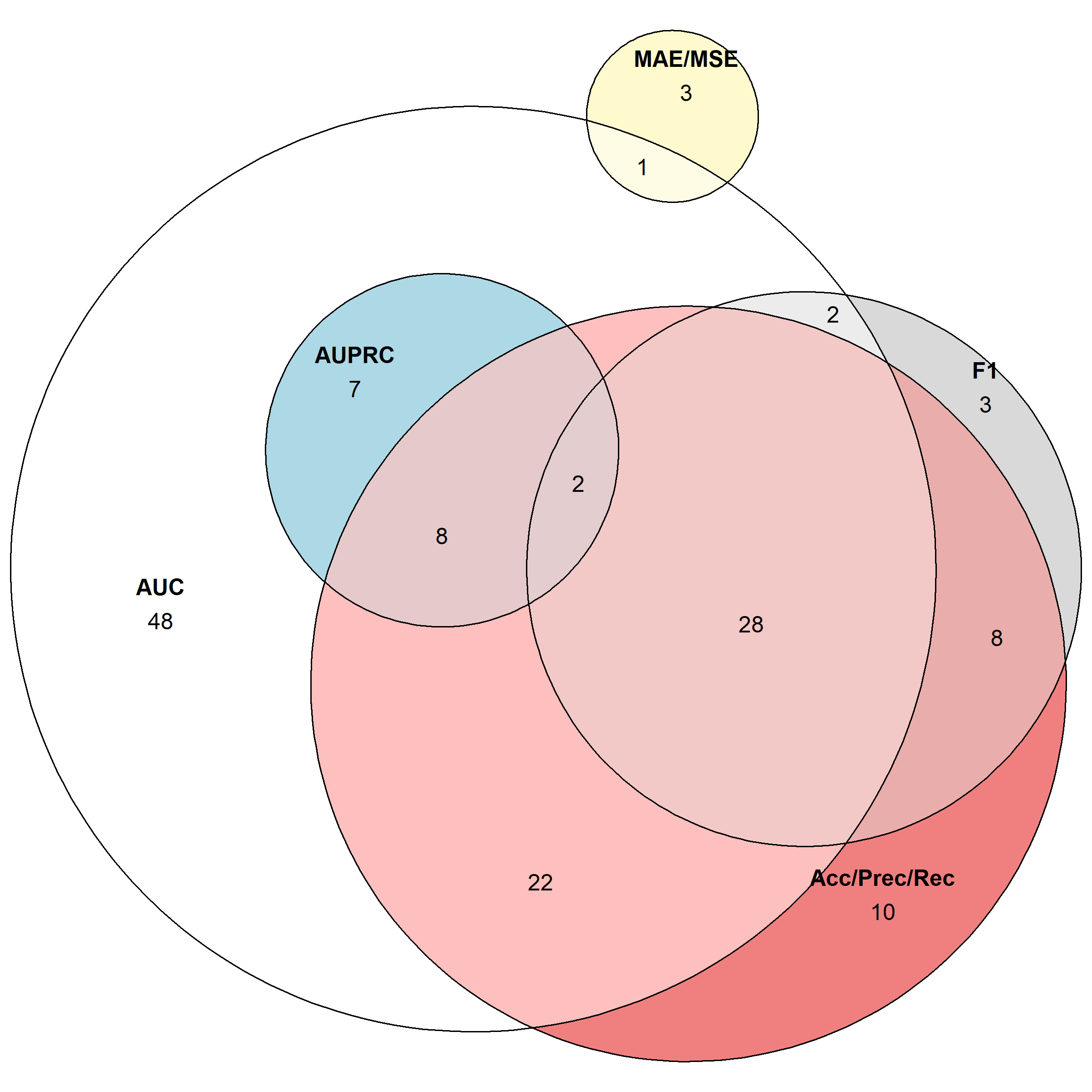

Supplement: ocac058_Supplementary_Data [file ocac058_supplementary_data.zip › FigureS4.png]
